# A Breath of Trouble: Unraveling the Impact of Air Pollution on Atrial Fibrillation

**DOI:** 10.3390/jcm13237400

**Published:** 2024-12-04

**Authors:** Anna Kurasz, Gregory Y. H. Lip, Sławomir Dobrzycki, Łukasz Kuźma

**Affiliations:** 1 Department of Invasive Cardiology, Medical University of Bialystok, 15-540 Bialystok, Poland; 2 Department of Cardiology, Lipidology and Internal Medicine, Medical University of Bialystok, 15-540 Bialystok, Poland; 3 Liverpool Centre for Cardiovascular Science at University of Liverpool, Liverpool John Moores University, Liverpool Heart and Chest Hospital, Liverpool L3 3AF, UK; 4 Danish Center for Health Services Research, Department of Clinical Medicine, Aalborg University, 9220 Aalborg, Denmark

**Keywords:** air pollution, AF, particulate matter, cardiovascular prevention, epidemiology, arrhythmia, health

## Abstract

Air pollution is a pervasive global challenge with profound implications for public health. This review explores the intricate relationship between air pollution and atrial fibrillation (AF), a prevalent cardiac arrhythmia associated with significant morbidity and mortality. Drawing on a comprehensive analysis of the existing literature, this review synthesizes current evidence linking various air pollutants, including particulate matter, nitrogen dioxide, ozone, and carbon monoxide, to the development and exacerbation of AF. The review delves into the role of air pollution as a global health issue alongside its specific sources, such as traffic-related emissions and industrial pollutants. It also examines the underlying mechanisms through which air pollution may contribute to the pathogenesis of AF, encompassing oxidative stress, inflammation, and autonomic nervous system dysregulation. In addition, it explores the impact of individual pollutants and the results of meta-analyses. It considers the results of vulnerable populations, including sex differences between the individuals and those with pre-existing cardiovascular conditions, who may be disproportionately affected. We also address critical research gaps in this area. Overall, air pollution has been increasingly recognized as a significant trigger for AF, with evidence linking exposure to particulate matter and gaseous pollutants to an increased incidence in short- as well as long-term exposure, highlighting the need for targeted public health interventions and further research to mitigate its cardiovascular impact.

## 1. Introduction

Atrial fibrillation (AF) is an increasing global health concern with a growing incidence and significant implications for morbidity and mortality from stroke, heart failure, dementia, and hospitalizations, as well as healthcare costs and poor quality of life [1,2,3]. Moreover, AF impacts the incidence and development of several conditions, such as cardiovascular disease, heart failure [4,5], and dementia [6], and is associated with multimorbidity and frailty [7,8,9].

Due to advances in medicine, pharmacotherapy, and the treatment of numerous chronic diseases, global life expectancy is increasing, collectively leading to a state in which AF prevalence is becoming a 21st-century cardiovascular epidemic [10]. According to estimates, in 2010, there were approximately 8.8 million adults with AF in the European Union, and it is projected that 17.9 million people will suffer from it by 2060 [11], leading to major impacts on healthcare costs [2]. Improved methods of diagnosis and treatment are enabling the progressively better control of AF, but better understanding and identification of risk factors may be the key to the overall prevention and management of the disease.

Over the years, numerous risk factors for AF have been identified, which can be categorized into non-modifiable and modifiable ones. Non-modifiable risk factors include sex, genetic factors, and advancing age, with the risk of AF increasing significantly as individuals become older [12,13]. Modifiable risk factors encompass lifestyle and health-related factors that can be controlled or managed. For example, hypertension, obesity, smoking, excessive alcohol consumption, and poorly managed diabetes are among the modifiable risk factors for AF, whilst conditions such as sleep apnea, physical inactivity, high cholesterol levels, stress, and dietary choices can contribute to an increased risk [13,14]. Given the heterogeneous and clinically complex phenotypes [15,16], the management of AF patients has moved towards a more holistic or integrated care approach [17], given the potential impact on improved outcomes [18].

While considerable attention has been devoted to understanding the clinical and lifestyle determinants of AF, emerging evidence suggests that environmental factors, particularly air pollution, may play a substantial role in the development and exacerbation of AF [12,19,20,21]. Air pollution, largely a consequence of industrialization and urbanization, has garnered extensive scrutiny for its adverse effects, mainly on respiratory and cardiovascular health [22,23]. However, its potential link to AF remains a topic of ongoing investigation. With the ever-increasing burden of air pollution and the concomitant rise in AF cases, understanding and addressing this nexus holds significant implications for both clinical management and public health interventions.

This review article aims to explore the relationship between air pollution and AF, addressing air pollution itself and potential mechanisms through which it may contribute to the onset and progression of AF. We will provide an in-depth analysis of the existing literature, discussing the key findings of studies not only on each pollutant influence but also the results of meta-analyses, which, to our knowledge, have not been performed collectively before for AF itself; we will also point to critical research gaps in this area.

## 2. Literature Search Methods

We reviewed the existing literature on air pollution’s influence on atrial fibrillation by conducting systematic searches of PubMed/Medline scientific database applying the following keywords: “air pollution” and “atrial fibrillation”. The last search was conducted on 4 January 2024, resulting in 112 hits. The titles and abstracts of all search results were assessed to fulfill the review criteria. Studies in languages other than English, case studies, analyses not including AF incidence, and research without full-text availability were excluded. For each article considered relevant, the reference list of eligible literature was browsed.

## 3. Air Pollution as a Global Health Issue

According to the estimates in the Global Assessment of the Burden of Disease from Environmental Risks by the World Health Organization (WHO), ambient air pollution accounts for 4.3 million deaths per year [24]. Data from the Global Burden Disease Study allowed for the analysis showing that in 2019, the number of air pollution-related deaths amounted to 6.67 million, which accounted for 11.62% of deaths globally [25]. The number of air pollution-related disability-adjusted life years (DALYs) was 21.33 million, accounting for 0.84% of global DALY in 2019 [25]. The magnitude of its effect is also reflected in the fact that air pollution currently ranks fourth among the major risk factors for global mortality among both men and women [26]. The economic aspect of this issue is also noteworthy, as the WHO European Region has estimated the total annual cost of health impacts and mortality from air pollution, including morbidity-related expenses, to be approximately USD 1.575 trillion [27].

Exposure to polluted air has been linked to a myriad of health problems, including respiratory diseases, which were first to be directly linked to smog exposure due to the natural process of air inhalation. Pollutants can induce asthma attacks and exacerbate patients’ existing symptoms [28]. Prolonged exposure can also contribute to the development of asthma and chronic obstructive pulmonary disease, especially in susceptible individuals [29,30]. The inhalation of polluted air is the second leading cause of lung cancer after tobacco smoking, contributing to approximately 20% of all lung cancer deaths [31]. Moreover, by changing the lung microbiome, air pollution may be a contributing factor to developing upper as well as lower respiratory tract infections [32].

Over the years, evidence of associations between air pollution and numerous cardiovascular diseases has also been established. Increased exposure to pollutants, especially particulate matter (PM), is associated with a higher incidence of acute coronary syndromes in the overall population and in the elderly [33,34]. A meta-analysis of more than 23 million participants showed a positive association between air pollution and an increased risk of hospital admission due to stroke [35]. Moreover, air pollution significantly accelerates climate change, with greenhouse gasses trapping heat in the atmosphere and causing global temperatures to rise; such indirect mechanisms also contribute to the deterioration of cardiovascular health [36].

The health consequences of breathing polluted air are numerous, wide-ranging, and devastating in many cases. Therefore, it is worth mentioning that under the weight of scientific evidence, the WHO released new, stricter air quality guidelines in 2021 regarding the pollutants on which the evidence of detrimental health effects from exposure has advanced the most [37].

### Air Pollution, Its Composition, Types, and Sources

Air pollution is a mix of hazardous substances that can be classified in a variety of ways, most commonly by their physical composition, further by their emission sources, and whether they are primary or secondary pollutants. Pollutants with the strongest evidence for public health concerns include particulate matter (PM) with a diameter equal or smaller than 2.5 (PM_2.5_) and 10 (PM_10_) microns, polycyclic aromatic hydrocarbons (PAHs) including benzo[a]pyrene (BaP), and gaseous pollutants consisting of carbon monoxide (CO), ozone (O_3_), nitrogen dioxide (NO_2_), and sulfur dioxide (SO_2_) [38]. Air pollution can originate either from anthropogenic (i.e., human activity), natural emissions sources or have a mixed origin.

**Figure 1:**
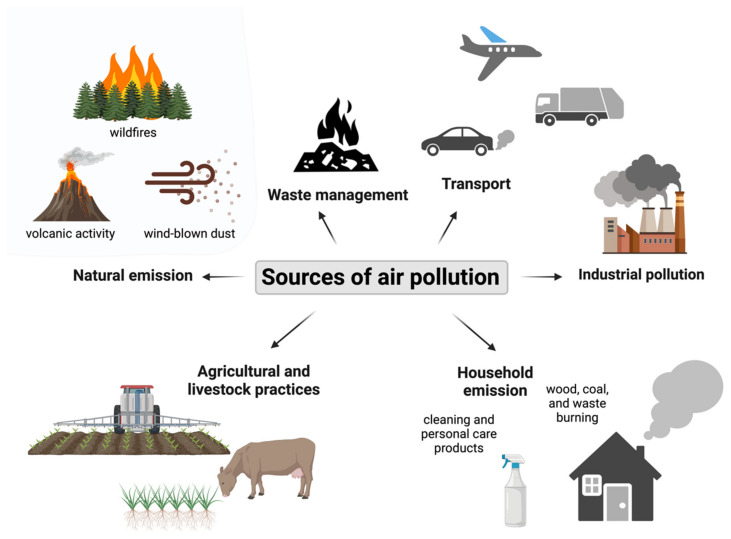
Sources of air pollution. (Created in Biorender.com).

The primary sources of environmental pollutants stem from extensive human activities, including emissions from cars, the operation of industrial machinery, power generation facilities, combustion engines, and inadequate waste management [39]. According to the European Environment Agency, in 2020, the primary source of particulate matter was energy consumption in the residential, commercial, and institutional sectors, with PM_10_ being responsible for 44% and PM_2.5_ for 58% of emissions [40]. The manufacturing industry and the road transport sector were also significant sources of PMs, with the addition of the road transport sector being the main source of nitrogen oxide emissions [40]. Moreover, several natural sources, such as volcanic eruptions, soil emissions, and wildfires, also play a role in shaping environmental conditions [Figure 1].

We can also distinguish several types of smog according to the different characteristics, sources, and compositions of pollution. Classical smog, widely known as ‘London smog’, is associated with the burning of sulfur-containing fuels and is prevalent in industrial areas. The incident of The Great London Smog of 1952, which lasted five days and caused the deaths of approximately 12,000 people, has led to air pollution being viewed as a major health risk factor [41]. Photochemical smog (‘Los Angeles Smog’) is a consequence of the interaction of sunlight with emissions from vehicles and industrial processes, resulting in high concentrations of O_3_ and NO_2_; it is often observed in urban areas with heavy traffic and sunny, warm weather [42]. ‘Polish smog’ is a form of air pollution that is mainly associated with low emissions from household heating using solid fuels (coal, wood, and often waste) and consists of high levels of PM_2.5_, PM_10_, and polycyclic aromatic hydrocarbons, especially BaP [43].

Due to their specific composition resulting in possible synergetic effects between particular pollutants, each of these types of smog has various detrimental health effects.

## 4. Contributing Mechanisms and Pathways Linking Air Pollution and Atrial Fibrillation

Although the exact mechanism of the effect of pollutants on the induction of AF is still debated, possible pathophysiological mechanisms that can worsen cardiovascular health and promote arrhythmia through a decrease in the arrhythmic threshold have been identified. We can distinguish the pulmonary activation of pro-inflammatory and pro-oxidant factors, leading to systemic inflammation (an increase in interleukin-6 and tumor necrosis factor-α); the transmural penetration of irritants through the pulmonary epithelium directly into the circulation and dysregulation of the autonomic nervous system [44,45].

The autonomic nervous system, comprising the sympathetic and parasympathetic branches, may play a pivotal role in regulating heart rate, triggering arrhythmias, and maintaining cardiovascular homeostasis [46]. Increased exposure to particulate matter is linked to elevated serum levels of stress hormones, including norepinephrine and epinephrine [45]. Changes in parasympathetic function lead to a reduction in the atrial effective refractory period, an increase in electrophysiological heterogeneity, early afterdepolarizations, and slowed conduction velocity in the atrium [47,48]. Vagal stimulation, as opposed to the sympathetic one, is antiarrhythmic; however, it can be proarrhythmic in the atria due to its different effects in the atria and ventricles [48].

## 5. Air Pollutants and Atrial Fibrillation

### 5.1. Particulate Matter Smaller than 2.5 Microns

In recent years, scientific interest from a focus on the impact of pollution on mortality, pulmonary disease, cancer, and ischemic heart disease has also begun to expand to include arrhythmias, with the majority of research analyzing the impact of particulate matter.

The impact of PM_2.5_ on AF incidence has been reported by multiple studies [Table 1]. However, one of the earliest research papers on this matter did not initially demonstrate a direct, significant relationship between PM_2.5_ and AF. In 2015, The Reasons for Geographic And Racial Differences in Stroke (REGARDS) Study performed an analysis focused on the possible influence of PM_2.5_ on premature atrial contractions (PACs) and AF in the short- (2 weeks) and long-term (1 year) [49]. Each 10 μg/m^3^ increase in short-term PM_2.5_ exposure was not associated with PACs or AF. An increase in long-term PM_2.5_ exposure also did not affect AF, but there was a significant increase in PAC occurrence.

**Table 1:** Studies from the review reporting outcomes of PM_2.5_ influence on AF occurrence.

First Author (Year) [Ref #]	n, Population	Pollutants Analyzed	Main Findings
O’Neal et al. (2015) [49]	28,502 participants from the Reasons for Geographic and Racial Differences in Stroke (REGARDS) Study.	PM_2.5_	Short-term PM_2.5_ (per 10 μg/m^3^) exposure was not associated with PACs (RR = 1.08, 95% CI = 0.99, 1.19) or AF (RR = 0.92, 95% CI = 0.84, 1.02). Increases in long-term PM_2.5_ per 10 μg/m^3^ were associated with PACs (RR = 1.35, 95% CI = 1.10, 1.65) but not AF (RR = 0.96, 95% CI = 0.79, 1.17)
Bunch et al. (2011) [50]	10,457 AF hospitalizations	PM_2.5_	Estimated associations between PM_2.5_ and AF hospitalizations from lag0 to lag21 were consistently positive and suggestive of risk, but they were not statistically significant
Liao et al. (2010) [51]	106 APACR Study participants equipped with personal PM_2.5_ monitors	PM_2.5_	PM_2.5_ adversely affects AF predictors on ECG with the maximal effects within 2 h after exposure
Link et al. (2013) [52]	176 patients with ICDs	PM_2.5_, black carbon, NO_2_, SO_2_, O_3_	26% increase in the odds of AF (95% CI 8–47%) for each 6.0 μg/m^3^ increase in PM_2.5_ in the 2 h prior to the event (*p* = 0.004)
Solimini et al. (2017) [19]	79,892 AF patients admitted to emergency departments	PM_2.5_, PM_10_, NO_2_	3% increase in AF (95% CI 1.4–4.7) for a 10 µg/m^3^ increase in PM_2.5_ at lag 0–1 day
Liu et al. (2018) [53]	100 individuals with cardiac implantable electronic devices	PM_2.5_, PM_10_, NO_2_, SO_2_, CO, O_3_	3.8% increase in AF risk (95% CI 1.4–6.2) for a 10 μg/m^3^ increase in PM_2.5_
Fang et al. (2021) [54]	15,171 AF patients	PM_2.5_, PM_10_, NO_2_, SO_2_, CO, O_3_	- 2.81% increase in AF risk (95% CI 1.44–4.20%) for a 10 μg/m^3^ increase in PM_2.5_- The results were robust after adjusting for SO_2_, NO_2_, CO, and O_3_
Yang et al. (2020) [55]	202,661 patients with cardiac arrhythmias	PM_2.5_	0.71% increase in AF (95% CI 0.18–01.25%, *p* < 0.01) per each 10 μg/m^3^ increase in PM_2.5_ at lag 0–2
Lee et al. (2019) [56]	670 patients diagnosed with AF for the first time	PM_2.5_, PM_10_, NO_2_, SO_2_, O_3_	- 22% increase in AF (95% CI 3–44%) for an IQR increase in PM_2.5_ (26.2 μg/m^3^) on the same day and a 19% (95% CI 0–40%) increase on the second day- Patients aged over 65 years with DM and hyperlipidemia were more vulnerable
Milojevic et al. (2014) [57]	Collective data from 3 large databases on MI, CVD, and CVD mortality	PM_2.5_, PM_10_, NO_2_, SO_2_, CO, O_3_	- PM_2.5_ exposure was not associated with acute hospitalizations due to AF- A 10th–90th centile change was associated with an increase of 21% (3.9 to 41%) in AF mortality
Andersen et al. (2021) [58]	23,528 Danish female nurses in a cohort with no prior diagnosis of AF	PM_2.5_, NO_2_	A 3.9 μg/m^3^ increase in 3-year mean PM_2.5_ concentration increased AF incidence before (HR 1.09; 95% CI 1.00–1.20) and after (1.08; 95% CI 0.97–1.19) adjustment for concurrent exposure to road traffic noise
Kwon et al. (2019) [59]	1137 AF emergency visits	PM_2.5_, PM_10_, NO_2_, SO_2_, CO, O_3_	- A 10 μg/m^3^ increase in PM_2.5_ was associated with an increase in emergency AF admissions by 4.5% at lag 3 (1.045; 95% CI, 1.002–1.089)- Long-term exposure to PM_2.5_ had no significant impact on AF (*p* = 0.83)
Jin et al. (2022) [60]	30 million Medicare beneficiaries aged > 65 years over	PM_2.5_, NO_2_, O_3_	- For each 1 μg/m^3^ increase in annual PM_2.5_, it resulted in an increased AF incidence (HR 1.0059, 95% CI 1.0054–1.0064)- Black people and patients with diabetes were more susceptible
Jones et al. (2023) [61]	11,249 older adult males	PM_2.5_	Long-term exposure to low-concentration PM_2.5_ was not associated with AF (HR 0.90, 95% CI 0.71–1.13)
Sun et al. (2023) [62]	1,374,423 individuals aged ≥ 35 years	PM_2.5_, PM_10_, O_3_	- An increase of 10 μg/m^3^ in PM_2.5_ (OR 1.031, 95% CI 1.010–1.053) was associated with AF prevalence- Diabetes and hypertension patients, females, and people aged < 65 years old were more vulnerable
Kim et al. (2019) [63]	432,587 subjects of the general population without AF diagnosis	PM_2.5_, PM_10_,	Long-term exposure of PM_2.5_ is associated with the increased incidence of new-onset AF (HR 1.179, 95% CI 1.176–1.183) for 10 μg/m^3^ increments

Abbreviations: 95% CI, confidence interval; AF, atrial fibrillation; CVD, cardiovascular disease; ECG, electrocardiogram; HR, hazard ratios; ICDs, implantable cardioverter-defibrillators; IQR, interquartile range; MI, myocardial infraction; PACs, premature atrial contractions; OR, odds ratio; RR, risk ratio.

Hence, these results suggest the potential role of long-term PM_2.5_ exposure in initiating supraventricular arrhythmias that are triggered by PACs but which do not sustain rhythm disorders such as AF. In 2011, a case-crossover study covering the period from 1993 to 2008 similarly did not observe the influence of PM_2.5_ on AF; even though estimated associations for the various lag structures were consistently positive and suggestive of some risk, they were not statistically significant [50].

The APACR Study equipped 106 participants with personal PM_2.5_ monitors in order to measure individual-level, real-time PM_2.5_ exposures during the same 24 h period and then evaluate it with a 24 h electrocardiogram (ECG) corresponding to that period. It was noted that PM_2.5_ adversely affects AF predictors on ECG, with the maximal effects experienced within 2 h after exposure; thus, PM_2.5_ may be indicative of greater susceptibility to AF [51].

This time window was reflected in the study by Link et al. [52], in which patients with implantable cardioverter–defibrillators (ICDs) were followed prospectively. While nonsignificant associations were found for PM_2.5_ in the 24 h after the exposure, the odds of AF increased by 26% (95% confidence interval [CI]: 8% to 47%) for each 6.0 mg/m^3^ increase in PM_2.5_ concentration in the 2 h prior to the event (*p* = 0.004) [52]. On the contrary, a 14-year time-series study reported that each 10 µg/m³ increase in PM_2.5_ resulted in a 3% (1.4–4.7) increase in the risk of AF emergency visits within 24 h of exposure with a stronger effect in patients aged >75 years [19]. Similar results were observed in another case-crossover design study, in which a 10 μg/m^3^ increase in PM_2.5_ was associated with a 3.8% (95% CI: 1.4–6.2) increase in the risk of AF occurrence [53]. Again, almost a 3% change in AF hospitalizations 2.81% (95% CI: 1.44%, 4.20%) resulted from a 10 μg/m^3^ increase in PM_2.5_, and the results were robust after adjusting for SO_2_, NO_2_, CO, and O_3_ [54]. The population of female patients and those aged >70 years were more vulnerable to this effect [54].

In an artificial intelligence-assisted analysis, the positive associations between PM_2.5_ and AF were noted for lag 0, 1, and 2 [55]. After controlling for the time trends, seasonality, day of the week, public holidays, and some of the weather conditions, the best-fitted lag model for predictions was lag 0–2 with a 0.71% (95% CI: 0.18–01.25%, *p* < 0.01) increase in arrhythmia per each 10 μg/m^3^ increase in PM_2.5_ [55]. A 5-year study from Taiwan also reported that the occurrence of AF was associated with PM_2.5_ exposure, even after a two-pollutant model was applied [56]. Moreover, in the detailed analysis, patients aged over 65 years and with comorbidities like diabetes mellitus and hyperlipidemia were more susceptible to the PM_2.5_ influence [56]. It is worth mentioning an analysis from three national databases in England and Wales that did not link PM_2.5_ exposure with acute hospitalizations due to AF; however, a 10th–90th centile change was associated with an increase of 21% (3.9 to 41%) in the AF mortality [57].

The influence of long-term exposure was assessed in a prospective analysis of a Danish female nurse cohort with no prior diagnosis of AF, which was followed for up to 14 years. The authors aimed to asses not only air pollution but also road traffic noise influence on AF [58]. A 3.9 μg/m^3^ increase in the 3-year mean PM_2.5_ concentration had an effect on the incidence of AF before and also after adjustment for concurrent exposure to road traffic noise (HR 1.09; 95% CI: 1.00, 1.20, and 1.08; 95% CI: 0.97, 1.19, respectively) [58]. A study from Soeul suggested that short-term exposure to PM_2.5_ triggers acute AF episodes; however, no evidence was found to link AF with long-term exposure [59]. Jin et al. performed an analysis of Medicare beneficiaries aged >65 years over the period of 2000 to 2016 and noted that long-term exposure to PM_2.5_ had an influence on the increased incidence of cardiovascular diseases, including AF, even at low pollutant concentration levels [60]. Also, black people and patients with diabetes were more susceptible to this adverse effect.

In contrast, in the Health in Men Study on older men, a significant association between long-term exposure to low-concentration PM_2.5_ and AF was not observed [61]. For example, a recent nationwide cross-sectional study in China found an increase in AF prevalence with each 10 μg/m^3^ increase in PM_2.5_ concentration (OR 1.031 [95% CI 1.010, 1.053]) [62]. Similarly to the previously mentioned study, this effect was stronger in patients with diabetes and additionally in females, people aged <65 years old, and those with hypertension [62]. In the Asian general population from the study covering 1,666,528 person-years, long-term PM_2.5_ exposure had an influence on increased incidence of new-onset AF (HR 1.179 [1.176–1.183] for 10 μg/m^3^ increments, *p* < 0.001), with a more profound effect found in obese male subjects aged above 60 years of age who had a history of hypertension or previous myocardial infarction [63]. The significance of the long-term PM_2.5_ impact can be reflected in the fact that an incident AF prediction model based on machine learning predicted the incidence of AF better than models without PM_2.5_ [64].

### 5.2. Particulate Matter Smaller than 10 Microns

PM_10_, right next to PM_2.5_, is the most frequently assessed pollutant in terms of detrimental health impacts. The impact of PM_10_ on AF incidence has been reported by multiple studies [Table 2].

One 2016 observational study from Italy reported that higher rates of acute AF hospitalizations were independently associated with a 1 μg/m^3^ increase in PM_10_ at lag 0-day (RR 1.008, 95% CI 1.003 to 1.012), and similar results were obtained using PM_10_ lag 3-day data [65]. A case-crossover study on the short-term influence of pollutants noted a 2.7% (95% CI: 0.6–4.8) increase in the risk of AF occurrence associated with a 10 μg/m^3^ increase in PM_10_ concentration [53].

**Table 2:** Studies from the review reporting outcomes of PM_10_ influence on AF occurrence.

First Author (Year) [Ref #]	n, Population	Pollutants Analyzed	Main Findings
Vaduganathan et al. (2016) [63]	6000 acute CV admissions	PM_10_	Acute AF hospitalizations were associated with a 1 μg/m^3^ increase in PM_10_ at lag0 (RR 1.008, 95% CI 1.003 to 1.012)
Liu et al. (2018) [52]	100 individuals with cardiac, implantable electronic devices	PM_2.5_, PM_10_, NO_2_, SO_2_, CO, O_3_	A 2.7% (95% CI: 0.6–4.8) increase in the risk of AF occurrence associated with a 10 μg/m^3^ increase in PM_10_ concentration
Dahlquist et al. (2020) [66]	8899 randomly selected 75-year-olds	PM_2.5_, PM_10_, NO_2_, O_3_	- PM_10_ impacted AF onset in the 12–24 h prior to the ECG recording with an OR of 1.10 (95% CI: 1.01–1.19) per 7.8 µg/m^3^ shift in pollutant concentration- People with diabetes and those overweight were more vulnerable
Solimini et al. (2017) [19]	79,892 AF patients admitted to emergency departments	PM_2.5_, PM_10_, NO_2_	- 1.4% increase in AF (0.7–2.3) for a 10 µg/m^3^ increase in PM_10_ at lag 0–1 day- The triggering effect of PM_10_ was 6% higher in individuals with previous CVD conditions within 24 h from exposure and 9% within lag0–5
Vencloviene et al. (2017) [67]	5361 patients who called the ambulance due to rhythm disturbances	PM_10_, CO	AF incidence at night was associated with PM_10_ at lag 2–5 days below the median (per IQR (7.31 μg/m^3^) increase: RR = 1.21, *p* = 0.002
Kim et al. (2019) [63]	432,587 subjects of the general population without AF diagnosis	PM_2.5_, PM_10_	Long-term PM_10_ exposure resulted in a 3.4% increase in AF incidents (HR 1.034, 95% CI 1.033–1.036) for 10 μg/m^3^ increments
Stockfelt et al. (2017) [68]	10,350 recruits from the PPS cohort and the GOT-MONICA cohort	PM_2.5_, PM_10_, NO_2_	AF was not positively associated with long-term exposure to both PM_2.5_ and PM_10_
Zhang et al. (2023) [69]	285,009 participants free of CVD at baseline from the UK Biobank	PM_2.5_, PM_10_, NO_2_	Positive associations between both PMs and the first admission, readmission, and total admission due to AF
Ma et al. (2023) [70]	401,251 participants without AF at baseline from UK Biobank	PM_2.5_, PM_10_, NO_2_	Participants exposed to high air pollutants levels and high genetic risk had an approximately 149.2% (PM_2.5_) and 181.7% (PM_10_) higher risk of AF compared to those with low air pollutants levels and low genetic risk

Abbreviations: 95% CI, confidence interval; AF, atrial fibrillation; CVD, cardiovascular disease; ECG, electrocardiogram; HR, hazard ratios; IQR, interquartile range; OR, odds ratio; RR, risk ratio.

Dahlquist et al. designed a study where almost nine thousand randomly selected 75-year-olds without previously known AF used home-based ambulatory 1-lead ECG measurements 2–4 times a day for 14 days [66]. The results of the analysis revealed that PM_10_ had a statistically significant association with AF onset in the 12–24 h prior to the ECG recording with an OR of 1.10 (95% CI: 1.01–1.19) per 7.8 µg/m^3^ shift in the pollutant concentration. Moreover, the effect was more pronounced among participants with diabetes and those who were overweight [66].

The association between polluted air and emergency room visits due to AF was explored by Solimini et al. in 2017, whereby a 10 µg/m^3^ increase in PM_10_ had an influence on AF emergency visits within 24 h of exposure with a higher effect on the male population [19]. The triggering effect of PM_10_ was 6% higher in individuals with previous CVD conditions within 24 h from exposure and 9% within lag 0–5 [19]. Researchers from Lithuania hypothesized that the influence of environmental factors on AF falls into a circadian variation, i.e., it varies during different periods of the day, but no significant association between PM_10_ exposure and emergency ambulance calls for paroxysmal AF in the afternoon was found [67]. However, the risk of those emergency calls at night–early in the morning (22:00–7:59) was associated with an increase in PM_10_ below the median of 2–5 days after exposure, and people aged above 65 years were more vulnerable [67].

In the previously mentioned study on the Asian general population, the incidence of new-onset AF was not only influenced by PM_2.5_ but also by long-term PM_10_ exposure, resulting in a 3.4% increase in AF incidents (HR 1.034 [1.033–1.036] for 10 μg/m^3^ increments, *p* < 0.001) [63]. The analysis of two Swedish cohorts from 1990 to 2011 (the PPS cohort and the GOT-MONICA cohort) assessed the long-term effects of particulate air pollution on various cardiovascular diseases, but AF was not positively associated with long-term residential air pollution exposure, including both PM_2.5_ and PM_10_ [68].

Another study focusing on the outcomes of air pollution exposure on cardiovascular disease occurrence with a median follow-up of 12 years noted consistently positive associations between not only both PMs but also NO_2_ and the first admission, readmission, and total admission due to AF [69]. A large prospective cohort study on 401,251 participants from UK Biobank without AF at baseline aimed to analyze the connection between air pollution, genetic susceptibility, and the risk of AF, whereby participants exposed to high levels of air pollutants and possessing a high genetic risk exhibited a significantly increased susceptibility to AF compared to individuals with exposure to low levels of air pollutants and a low genetic risk [70]. Those from the former group had a 181.7% higher risk associated with PM_10_ exposure in comparison to the latter, and PM_10_ increased this risk the most among the investigated pollutants (149.2%—PM_2.5_ and 170.2%—NO_2_) [70]. However, in the previously mentioned Seoul study, among the 1137 emergency visits due to AF and 1903 patients who developed AF at a median follow-up of 9.5 years, PM_10_ was not associated with either a short- or long-term influence on AF [59].

### 5.3. NO_2_ and Other Gaseous Pollutants

Studies reporting outcomes of NO_2_ and other gaseous pollutants on AF occurrence are summarized in Table 3.

The influence of short-term air pollution (PM_2.5_, PM_10_, SO_2_, NO_2_, and O_3_) on emergency hospital admissions was investigated in a case-crossover analysis of the MINAP database [57]. Of all the pollutants analyzed, only NO_2_ was associated with a higher risk of emergency AF admissions; a 10th–90th centile change was associated with a 2.8% (95% CI 0.3–5.4%) increase in AF cases [57].

Saifipour et al. aimed to assess the connection between air pollution and AF hospitalizations in patients with ventricular response above and below 90 beats per minute in Iran, but the only significant relationship observed in the analysis was the one between NO_2_ exposure and hospitalization due to AF in patients with rapid ventricular response [71]. One study focused particularly on NO_2_ and observed that each 10 μg/m^3^ increase in NO_2_ was associated with an increased risk of AF on the day of exposure [72]. Furthermore, the female population had a higher risk of AF on the day of exposure (OR 1.051; 95% CI 1.019–1.083) as well as on the following day (1.050; 95% CI 1.019–1.083) [72]. Females were also more vulnerable to NO_2_ triggering effects on AF emergency hospitalizations [19].

**Table 3:** Studies reporting outcomes of NO_2_ and other gaseous pollutants on AF occurrence.

First Author (Year) [Ref #]	n, Population	Pollutants Analyzed	Main Findings
Zhang et al. (2023) [69]	285,009 participants free of CVD at baseline from the UK Biobank	PM_2.5_, PM_10_, NO_2_	Positive associations between NO_2_ and the first admission, readmission, and total admission due to AF
Ma et al. (2023) [70]	401,251 participants without AF at baseline from UK Biobank	PM_2.5_, PM_10_, NO_2_	Participants exposed to high air pollutant levels and high genetic risk had an approximately 170.2% (NO_2_) and 157.2% (NO_x_) higher risk of AF compared to those with low air pollutants levels and low genetic risk
Kwon et al. (2019) [59]	1137 AF emergency visits	PM_2.5_, PM_10_, NO_2_, SO_2_, CO, O_3_	A 10 μg/m^3^ increase in PM_10_ was not associated with either a short- or long-term influence on AF
Milojevic et al. (2014) [57]	Collective data from 3 large databases on MI, CVD, and CVD mortality	PM_2.5_, PM_10_, NO_2_, SO_2_, CO, O_3_	A 10th–90th centile change in NO_2_ resulted in a 2.8% (95% CI 0.3–5.4%) increase in AF emergency cases
Halldorsdottir et al. (2022) [72]	13,664 individuals with emergency hospital visits for heart disease diagnoses	M_2.5_, PM_10_, NO_2_, SO_2_	- Each 10 μg/m^3^ increase in NO_2_ was associated with an increased risk of AF on the day of exposure- The female population had a higher risk of AF on the day of exposure (OR 1.051; 95% CI 1.019–1.083) and on the following day (1.050; 95% CI 1.019–1.083)
Cakmak et al. (2014) [73]	8595 subjects who had 24 h ambulatory Holter monitoring	PM_2.5_, NO_2_, O_3_	- An IQR in NO_2_ was linked to a 4.39% (−0.15, 9.15) increase in the duration of atrial fibrillation among individuals aged 50 or below and a 7.1% (0.24, 14.5) increase among the male sex- A 6% increase in time in AF associated with NO_2_ during the cold season was found (October–March)
Zhou et al. (2022) [74]	8307 outpatient visits for AF	NO_2_	- A 10 μg/m^3^ increase in NO_2_ at lag 03 was related to an elevation of 5.59% (95% CI: 2.67%, 8.51%) in daily outpatient visits for AF- No gender or age difference on the effect of NO_2_- A stronger association was observed in cold seasons (October to March) than in warm seasons
Vencloviene et al. (2017) [67]	5361 patients who called an ambulance due to rhythm disturbances	PM_10_, CO	CO significantly increased the risk of emergency calls for AF before noon and at night or early in the morning
Xue et al. (2023) [75]	190 115 patients with acute onset of symptomatic arrhythmia	PM_2.5_, PM_10_, NO_2_, SO_2_, CO, O_3_	A 3.4% increase in the odds of AF onset associated with an IQR increase in the NO_2_ concentration (95% CI 1.8–5.1%), with NO_2_ having the strongest impact on arrhythmias out of all the analyzed pollutants
Han et al. (2023) [76]	10,325 in-hospital patients whose ECG reports indicated AF	PM_2.5_, PM_10_, NO_2_, SO_2_, O_3_	- NO_2_ increased the risk of daily visits of AF recorded by ECG and the maximum OR on the day of exposure (1.0381; 95% CI 1.0135–1.0634)- Protective effect of O_3_ on AF occurrence on the day of exposure (0.9972; 95% CI 0.9949–0.9995) as well as after 48 h (0.9869; 95% CI 0.9791–0.9948)
Hart et al. (2021) [77]	83,117 postmenopausal women from the Women’s Health Initiative Cohort	PM_2.5_, PM_10_, NO_2_, SO_2_	NO_2_ was associated with the risk of AF (HR 1.18, 95% CI 1.13–1.24)
Monrad et al. (2017) [78]	57,053 people aged 50–64 years old	NO_2_	A 10 μg/m^3^ higher 10-year time-weighted mean exposure to NO_2_ was associated with an 8% higher risk of AF (incidence rate ratio: 1.08; 95% CI 1.01–1.14)
Kwon et al. (2019) [59]	1137 AF emergency visits	PM_2.5_, PM_10_, NO_2_, SO_2_, CO, O_3_	No association between the occurrence of AF episodes and short- or long-term exposure to any gaseous pollutants
Andersen et al. (2021) [58]	23,528 Danish female nurses’ cohort with no prior diagnosis of AF	PM_2.5_, NO_2_	Long-term exposure to NO_2_ had no influence on AF occurrence (HR 1.03, 95% CI 0.98, 1.08); this result persisted before and after adjustment for road traffic noise

Abbreviations: 95% CI, confidence interval; AF, atrial fibrillation; CVD, cardiovascular disease; ECG, electrocardiogram; HR, hazard ratios; IQR, interquartile range; MI, myocardial infarction; OR, odds ratio.

An analysis performed between 2004 and 2009 in Canada on 8595 subjects who had 24 h ambulatory Holter monitoring also confirmed the detrimental influence of NO_2_ [73]. An interquartile increase in NO_2_ was linked to a 4.39% (−0.15, 9.15) increase in the duration of atrial fibrillation among individuals aged 50 or below and a 7.1% (0.24, 14.5) increase among the male sex [73]. Additionally, the analysis revealed that there was a 6% increase in time for AF associated with NO_2_ during the cold season (October–March) [73]. The seasonal results of the aforementioned analysis were also reflected in a hospital-based study in northwestern China, whereby increased NO_2_ levels had an influence on increased AF outpatient visits; there were no gender or age differences in the NO_2_ effect; however, a stronger association was observed in the cold seasons (October to March) than in warm seasons [74].

As for circadian variation, similar to the results of PM_10_ mentioned before, CO did not have an impact on emergency ambulance calls for paroxysmal AF in the afternoon; however, there was a significantly increased risk of emergency calls for AF before noon and at night or early in the morning [67]. In addition, Xue et al. analyzed hourly air pollution exposure and the onset of symptomatic arrhythmia in their case-crossover study on an individual level in 322 Chinese cities, covering data from 2025 hospitals, finding a 3.4% increase in the odds of AF onset associated with an IQR increase in the NO_2_ concentration (95% CI 1.8–5.1%), with NO_2_ having the strongest impact on arrhythmias out of all the analyzed pollutants [75].

Han et al. designed a case-crossover study in which 4933 male and 5392 female patients whose ECG reports indicated AF were enrolled over the 4-year period. NO_2_ increased the risk of daily visits of AF recorded by ECG and the maximum OR on the day of exposure (1.0381; 95% CI 1.0135–1.0634). What is interesting is that O_3_ showed a protective effect on AF occurrence on the day of exposure (0.9972; 95% CI 0.9949–0.9995) as well as after 48 h (0.9869; 95% CI 0.9791–0.9948), and no effect of PMs and SO_2_ was noted [76].

Long-term air pollution exposure was also assessed in postmenopausal women from the Women’s Health Initiative Cohort, covering over 660,236 person-years of follow-up. In this study, NO_2_ was associated with the risk of AF (HR 1.18, 95% CI 1.13–1.24), contrary to PMs and SO_2_ [77]. Furthermore, long-term exposure to traffic-related air pollution was analyzed in a cohort of 57,053 people 50–64 years old who were enrolled in 1993–1997. A 10 μg/m^3^ higher 10-year time-weighted mean exposure to NO_2_ was associated with an 8% higher risk of AF [78]. On the other hand, in a time period analysis over the years 2007–2015 on the nationwide cohort from the Korean general population, no association was found between the occurrence of AF episodes and short- or long-term exposure to any gaseous pollutants, and only the effect of particulate matter was noted [59]. In the Danish Nurse Cohort, long-term exposure to NO_2_ had no influence on AF occurrence; this result persisted before and after adjustment for road traffic noise [58]. More information on the impact of other gaseous pollutants can be found in Section 5 and Section 5.4.

### 5.4. Impact of Pollution on Patients with Implantable Cardioverter-Defibrillators

Patients with ICDs constitute a specific population in terms of assessing the impact of air pollution on arrhythmia occurrence, although they are burdened with cardiac diseases that have led to the implantation of the ICD, such as myocardial infarction and heart failure, which are strongly associated with AF onset. Hence, ICD patients might be prone to stimulation by air pollutants.

A time-stratified case-crossover study from 2020 analyzed 584 AF episodes from 91 participants with intracardiac devices [79]. Short-term increases in PM_2.5_ concentration in a low-pollution-level environment resulted in an increased risk of AF episodes. No effect of PM_10_ nor NO_2_ was observed, but results were suggestive of O_3_ influence during the warm season [79]. Researchers from Italy noted an association between daily levels of both PM_2.5_ (OR 1.80; 95% CI 1.34–2.40) and PM_10_ (OR 2.48; 95% CI 1.44–4.28) and AF occurrence for an increase of 50 µg/m^3^ above the WHO threshold [80].

An analysis of short-term exposure to air pollution on AF occurrence found a significantly positive effect of only PM_2.5_ and PM_10_ and not any of the gaseous pollutants [53]. Conversely, in a case-crossover study from Boston, a two-fold increase in the risk of paroxysmal AF episodes was associated with an increase in mean ambient O_3_ concentration in the concurrent hour [81]. Associations for PM_2.5_ and NO_2_ exposure were positive but not statistically significant [81].

### 5.5. Results of Meta-Analyses

In 2016, a meta-analysis containing only four studies was published that concluded a statistically significant association between AF occurrence and all analyzed pollutants (PM_2.5_, SO_2_, NO_2_, O_3_, and CO) [82]. Five years later, Chen and colleagues included 18 studies in their analysis [83]. Their results suggest that short-term exposure to PM_2.5_, SO_2_, and NO_2_ increases AF incidence; the effects varied significantly in sub-analyses by region, gender, outcome, and age. Moreover, long-term exposure to all air pollutants (PM_2.5_, PM_10_, SO_2_, NO_2_, CO), except O_3_, was associated with increased AF incidence in a healthy population [83].

Yue et al. also performed a meta-analysis of 18 studies but focused on short- and long-term air pollution influence on AF occurrence in the general population [84]. For each increment of 10 μg/m^3^ in the short term, the combined OR of AF onset was 1.01 for PM_2.5_ exposure (95% CI 1.00–1.02) and 1.03 for PM_10_ (1.01–1.05). All gaseous pollutants (SO_2_, NO_2_, CO, O_3_) also had a significant effect on AF prevalence in the short term. The long-term-exposure effect on AF prevalence was noted for each increment of 10 μg/m^3^ in the PM_2.5_ (OR 1.07; 1.04–1.10) and PM10 concentration (1.03; 1.03–1.04), as well as for a 10 ppb increment in the NO2 concentration (1.02; 1.00–1.04) [84].

A 2021 meta-analysis focused on PM_2.5_ exposure and AF in older adults (average age > 50 years old) was carried out [85]. Results from 16 observational studies revealed a statistically significant influence of PM_2.5_ on increased AF incidence (pooled OR 1.11; 95% CI: 1.03–1.19), and this effect was stronger in areas with higher levels of the pollutant (≥25 μg/m^3^). [85]. As for the meta-analysis of air pollution outcomes in patients with ICD, Yue et al. concluded that short-term PM_2.5_ exposure had an influence on the occurrence of AF episodes with the combined OR of 1.24 (95% CI 1.00–1.53) for each 10 μg/m^3^ increment in PM_2.5_ concentration [86].

## 6. Challenges and Future Directions

There is a need for more work analyzing air pollution as a whole; so far, most of the studies focused on atrial fibrillation have analyzed only selected pollutants, which allows only a partial look at the spectrum of the impact of air pollution. Moreover, there is a clear gap in research on the arrhythmogenic effects of BaP, although its harmful effects on the cardiovascular system have been demonstrated in numerous studies [87].

Exposure to polyaromatic hydrocarbons is associated with cardiac arrhythmias, and benzo(a)pyrene belongs to the PAHs group, which further prompts the expansion of future analysis to include BaP [88]. It seems that there is a scarcity of studies that demonstrate in detail the impact of pollutants on AF incidence in specific groups, with a breakdown not only by age and gender but also by factors such as place of residence and socio-economic status. The results of such analyses will help improve clinical patient care and precisely expand primary and secondary prevention methods.

Together with advances in technology, the ability to detect atrial fibrillation in patients has increased, which is reflected in current guidelines [89]. Unlike ICDs, wearable devices can be used in the general population, giving the possibility of screening or detecting AF early along with the precise overlay of environmental data. Therefore, it is worth highlighting emerging studies using smart devices to investigate the influence of polluted air on AF onset, which may serve as a determinant for the methodology of future research [90].

While we wait for changes in global air quality, there are some steps that can be taken in an attempt to individually reduce one’s risk of AF induced by air pollution. Indoor air quality can be improved using air purifiers and personal protective equipment like masks with HEPA filters, which mitigate exposure to outdoor pollution. Furthermore, adopting a healthy lifestyle and managing pre-existing cardiovascular conditions can contribute to increased cardiovascular (CV) resilience. In a recent study by Zhang et al., ideal body mass index and blood pressure were associated with a decrease of 11.57% and 11.46% in AF cases associated with PM_2.5_ [21]. Participants with better CV health had a smaller risk of AF incidence than those with low CV health [21].

To address the role of air pollution in triggering AF, public health policies should prioritize stricter air quality standards and expand monitoring networks and emission reduction initiatives targeting transportation and industry. Additionally, implementing community-level protections, public education campaigns, and integrating environmental exposure assessments into cardiovascular care can mitigate the risks for at-risk populations. Further research and technological innovations, such as wearable devices and AI-driven prediction tools, are crucial for advancing preventive strategies and reducing the burden of AF linked to air pollution.

## 7. Conclusions

This comprehensive narrative review demonstrates the detrimental effects of air pollution, distinguishing individual pollutants and their potential to induce AF in short- as well as long-term exposure. It also highlights the need to continuously reduce air pollution in order to improve population health by lowering the AF burden. More in-depth research is needed, focusing on pollutants that have been under-studied so far and on the impact of specific types of smog in particular areas to enable the implementation of local public health strategies tailored to the environment.

Future studies should focus on standardized research protocols, global collaborative initiatives, and the integration of innovative technologies to address air pollution challenges. This will provide a more comprehensive understanding of the intricate relationship between air pollution and AF as well as strengthen the weight of scientific evidence, which should result in the inclusion of air pollution as a risk factor for AF in management guidelines. Finally, more attention should be paid to the importance of preventive education among patients directed at modifiable risk factors such as air pollution.
